# Natural infection by *Leishmania infantum* in the *Lutzomyia longipalpis* population of an endemic coastal area to visceral leishmaniasis in Brazil is not associated with bioclimatic factors

**DOI:** 10.1371/journal.pntd.0007626

**Published:** 2019-08-26

**Authors:** Tiago Feitosa Mota, Orlando Marcos Farias de Sousa, Yuri de Jesus Silva, Lairton Souza Borja, Bruna Martins Macedo Leite, Manuela da Silva Solcà, Djalma Alves de Melo, Claudia Ida Brodskyn, Edelberto Santos Dias, Patrícia Sampaio Tavares Veras, Deborah Bittencourt Mothé Fraga

**Affiliations:** 1 Instituto Gonçalo Moniz—Fundação Oswaldo Cruz, Salvador, Brazil; 2 Escola de Medicina Veterinária e Zootecnia—Universidade Federal da Bahia, Salvador, Brazil; 3 Centro de Controle de Zoonoses de Camaçari, Camaçari, Brazil; 4 Instituto René Rachou—Fundação Oswaldo Cruz, Belo Horizonte, Brazil; 5 Instituto Nacional de Ciência e Tecnologia de Doenças Tropicais—INCT-DT, Salvador, Brazil; Saudi Ministry of Health, SAUDI ARABIA

## Abstract

Visceral leishmaniasis (VL) is a zoonosis caused by the protozoan *Leishmania infantum* and in Brazil is transmitted mainly by the bite of *Lutzomuyia longipalpis* sand flies. Data about the presence, distribution, natural infection rate, seasonal and monthly dynamics of the vector population are important for optimizing the measures to control VL in endemic areas. This study aimed to identify sand fly fauna in an endemic area for VL to detect the prevalence of *L*. *infantum* infection in the *Lu*. *longipalpis* population and to elucidate the influence of bioclimatic factors on the monthly fluctuations of this vector. HP light traps were monthly set in the intradomicile and peridomicile of residences located in the central and beachfront areas of Camaçari, a VL endemic area. The sand fly collection was conducted in two periods: i) period 1—between December 2011 and November 2012 and ii) period 2—August 2014 and July 2015. Sand fly species were identified and detection of *L*. *infantum* infection by qPCR was performed in pools of female *Lu*. *longipalpis*. For the first time, the parasite load of positive pools was correlated with the number of *Lu*. *longipalpis* captured per month in both periods. Correlation analyses between the monthly fluctuation of the sand fly population and bioclimatic indices of the municipality in both collection periods were also performed. In both evaluated periods, more than 98% of the collected sand flies were *Lu*. *longipalpis*, confirming the predominance of this species in the region. It was captured mostly in the beachfront area in all months evaluated (99%). For the period 1, *Leishmania* DNA was detected in 81% of tested pools representing a minimal infection rate of 9.6%. In the period 2, 40% of the pools were positive with a minimal infection rate of 10.2%. Infected sand flies were only detected in the beachfront area in both periods. The parasite load was low and did not vary in the evaluated months despite the number of collected sand flies. No correlation was observed for climatic factors in both areas of Camaçari. These findings emphasize the high risk of *Leishmania* transmission in Camaçari regardless of the season and that other factors, aside from bioclimatic elements, are influencing the sand fly population monthly fluctuation.

## Author summary

Visceral leishmaniasis (VL) is a parasitic disease transmited by a sand fly vector. VL is an important public health issue in Brazil occurring in all its regions with increasing incidences in big urban centers. In the State of Bahia, VL has spread to new hotspots in the last decade reaching the coastal area to the north of Bahia State capital, Salvador. This region underwent several environmental changes due to a rapid urbanization and construction of touristic and vacation complexes. Our investigation represents the first entomological study conducted in Camaçari. We evaluated the prevalence of the parasite infection in the sand fly population in two periods, between December 2011 and November 2012 and between August 2014 and July 2015. Our findings indicate that VL transmission occurs during the whole year and support the idea that the vector is not sensible to bioclimatic variations in Camaçari. Additionally, the maintenance of the parasite infection in the vector regardless of the season, indicates that the disease is well stablished in Camaçari, especially in the coastal area. These results emphasizes the urgency for a rigorous entomological and epidemiological survey aiming to guide control strategies to decrease VL cases.

## Introduction

Visceral leishmaniasis (VL) is an infectious disease considered to be neglected by the World Health Organization, mainly affecting poor regions in developing countries [1]. Previously in Brazil, likewise in other countries, the occurrence of VL was limited to rural areas and small urban localities [2,3]. However, the disease underwent an urbanization process with high incidence in big urban centers such as Belo Horizonte (Minas Gerais State), Campo Grande (Mato Grosso do Sul State), Palmas (Tocantins State) and Araçatuba (São Paulo State) [2, 4, 5, 6]. Nowadays, approximately 70% of VL cases occur in urban areas [3]. In the last decade, canine and human cases of VL were diagnosed in the southern States of Brazil, considered non-endemic until 2008 [7, 8, 9, 10, 11].

In the State of Bahia, several studies were previously conducted, aiming to identify the phlebotomine sand fly species present in municipalities endemic for VL, such as Jacobina, Jequié and Camaçari [12, 13, 14]. Nevertheless, these studies are old, lacking in the literature updated data on the sand fly species present in Bahia. Additionally, little is known about the fauna of vectors in urban and peri-urban areas of municipalities in Bahia where the transmission is considered intense, as occurs in Camaçari municipality [15, 16]. Thus, entomological surveys should be conducted in these areas to elucidate which sand flies species are present, the density of the vector population, its distribution, and seasonality. Studies to evaluate the natural infection rate of *Lu*. *longipalpis* with *L*. *infantum* and to elucidate the seasonal and monthly population dynamics are also necessary. Such studies should be performed employing entomological monitoring and evaluation of climatic factors’ influence on the sand fly population. These evaluations are essential for the optimization of effective control measures for VL to be implemented in endemic areas [17, 18, 19, 20, 21, 22, 23].

In the evaluation of captured sand flies, the classical microscopic analysis and culture methods have been used to detect natural infection in sand flies, these techniques were performed in several studies [24, 25, 26, 27, 28]. However, the low sensitivity of these methods, the operational difficulty and delay to deal with a large number of samples and the low prevalence of *L*. *infantum* infection in the vectors in endemic areas limit the detection of parasites in sand flies [29, 30, 31]. In this context, a series of PCR assays to detect *Leishmania* DNA has been applied in studies to determine the natural infection rates in sand flies in the last years. Such assays aim to replace the aforementioned classical methods to increase the sensitivity and specificity in the detection of *Leishmania* parasites in the vectors [32, 33, 34, 35, 36, 37].

The aim of the present work was to identify the sand fly fauna present in Camaçari in two collection periods and to determine, for the first time, the prevalence of *Leishmania* infection in the sand fly population of this municipality during the whole year and, additionally, to elucidate the influence of bioclimatic factors on the population dynamics of these insects.

## Materials and methods

### Ethics statement

All experimentation involving canine specimens was performed in compliance with Brazilian federal law for animal experimentation (Law 11794), in conformity with the guidelines for animal experimentation established by the Oswaldo Cruz Foundation (FIOCRUZ), and in accordance with the procedures described in the Brazilian Ministry of Health’s manual for the VL surveillance and control. The present study received approval from the Institutional Review Board (CEUA protocol no. 015/2009, 017/2010 and 007/2013) of the Institute Gonçalo Moniz in Bahia, Brazil (IGM–FIOCRUZ/BA). Dog owners who agreed to participate in this study signed a form of Free, Prior and Informed Consent (FPIC).

### Study area and selection of households

**Fig 1:**
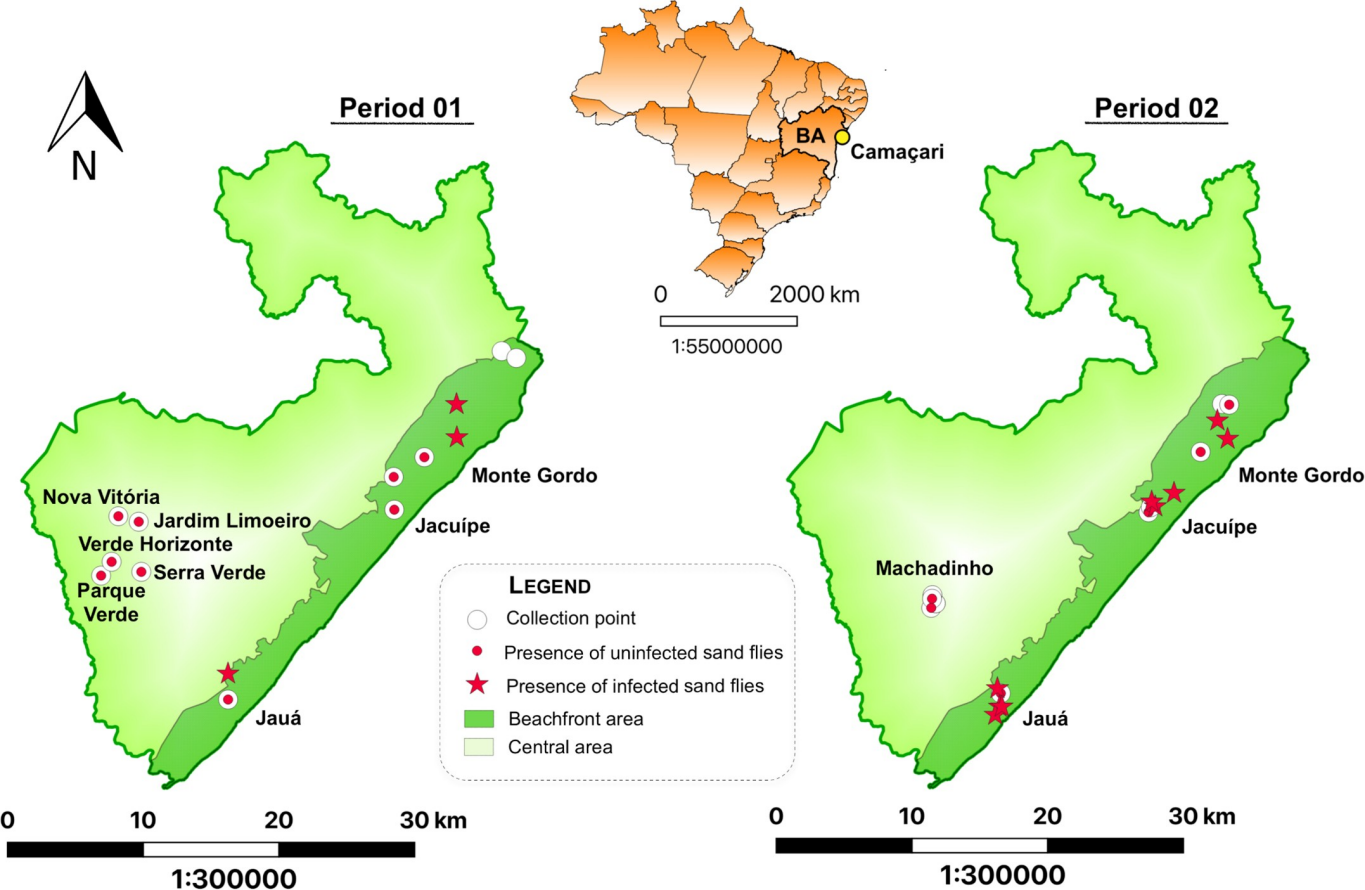
Map illustrating the localization of the Municipality of Camaçari in the state of Bahia, Brazil, and the sand fly collection points in the two one-year periods. Maps show the spatial distribution of sand fly collection points (white dots) in each period: the red dot indicating the presence of sand flies and the red star indicating the presence of infected sand flies. The beachfront area is represented as dark green (close to the coastal strip). Source: QGIS 2.14.3-Essen.

The study was conducted in the municipality of Camaçari, considered the biggest in the metropolitan region of Salvador, located at latitude 12º52’30” and longitude 38º28’52”. The climate in Camaçari is hot and humid, with minimum temperatures above 18°C, terrain formed by fluvial-marine and coastal plains and pre-coastal vegetation. Camaçari is an endemic area for visceral leishmaniasis and during the study two human cases occurred in Monte Gordo, a city located in the neighborhood of VL area evaluated [38]. For the entomological study, Camaçari was stratified into two areas: beachfront (close to the coastal strip) and central (far from the coastal strip) (Fig 1).

The collections of the insects were conducted in two one-year periods: i) period 1 from December 2011 to November 2012 and ii) period 2 –from August 2014 to July 2015. Captures were performed in neighborhoods presenting a high prevalence of canine visceral leishmaniasis (CVL). In period 1, the sand fly captures were done in 12 households (collection sites) distributed in three neighborhoods in the beachfront area: Monte Gordo (3 houses), Barra de Jacuípe (2 houses) and Jauá (1 house), and six neighborhoods in the central area (1 house in each one): Parque Real Serra Verde, Verde Horizonte, Nova Vitória, Parque Verde II, Phooc III, and Jardim Limoeiro. In period 2, the captures were performed in four neighborhoods: Machadinho in the central area, and Jauá, Barra de Jacuípe and Monte Gordo in the beachfront area. In each one, four households were selected, making a total of 16 collection sites.

In both collection periods, the residences that presented higher chances of the collection were selected, based on the presence of natural breeding sites for sand flies. The presence of abundant organic matter, areas with high humidity, animal shelters close to residences, visible tree roots and holes in stones or soil in the peridomicile were considered attractive factors for the presence of sand flies. Information regarding the occurrence of VL and/or CVL cases in the residence and neighboring houses, notified by the municipality’s epidemiological survey in the last five years or informed by the residents were also taken into account.

### Collection, identification and design of sand fly pools

The investigations were carried out with light traps, which were placed every month until 12 months, at the intradomicile and peridomicile of households for three consecutive nights. The traps remained on between dusk (17:00h) and dawn (6:00h) of the next day when the insects were captured and gathered. The captured sand flies were identified as described by Young and Duncan [39], in which the males were clarified and mounted on slides while for the females, only the head and the last two abdominal segments were mounted in Berlese liquid, according to a modified technique from Langeron [40]. The rest of the female bodies were stored in 6% dimethyl sulfoxide (DMSO) and kept at—70°C until the design of pools and DNA extraction.

Each pool was composed by two to ten female fragments of the species *Lu*. *longipalpis* collected in the same neighborhood, month and household. A total of 674 *Lu*. *longipalpis* non-fed females were used to build 74 pools in period 1, and 166 females were divided into 42 pools in period 2.

### Genomic material extraction

The pools of *Lu*. *longipalpis* females were macerated and homogenized in 60μL of lysis buffer (100mM Tris-HCL, 100 mM NaCl, 25 mM EDTA, 0,5% SDS, pH 8,0) and 10μL of proteinase k at 1 mg/L (Invitrogen Life Technologies, Carlsbad, CA, USA). The macerated material was incubated in a water bath for 16 to 18 hours at 37°C. The DNA was purified using phenol-chloroform technique and then precipitated with ethanol following the protocol described by Michalsky et al. (2002). The DNA samples were resuspended in 50μL TE buffer (Tris-EDTA 1X), and their concentrations were measured by digital spectrophotometry (Nanodrop ND-1000 Thermo Scientific, Wilmington, USA). Lastly, the samples were aliquoted at 20 ng/μL and stored at -20°C until their usage for qPCR.

### qPCR assay

The amplification of the gene control of *Lu*. *longipalpis* (GenBank AF446142; [41]) was used to normalize the DNA concentration of *L*. *infantum* present in the sand fly pool samples, as well as to assure that the negative results did not occur due to DNA degradation or the presence of inhibitors. For the amplification of the gene control, forward (Fleb-F) and reverse (Fleb-R) primers, 5'-AATTTCTTTTCCTTAGGACCATCGA-3' and 5'-TAGGACATCTTCGGAAAATTGTTG-3' respectively, were used together with a fluorogenic probe 5'-AMTCCTCASAGTCTTTGACTCCACGTTGGTT-3' aiming for the *Lu*. *longipalpis* periodicity gene which does not amplify *Leishmania* genomic DNA. The temperature conditions for the reaction were 50°C for 2min, 95°C for 10min and 40 cycles of 95°C for 15s and 60°C for 1min. A standard curve for the gene control was used for each reaction using DNA from pools of colony-reared males. For each sample, the quantity of gene control amplified DNA was determined by the comparison between the samples’ and curves’ Ct values. Only the samples with amplification for periodicity gene were used, to determine the *Leishmania* infection rate.

qPCR assays aiming to determine the quantity of the parasite’s DNA in the pools of captured female sand flies were based on a protocol described by Francino et al. [42] adapted by Solcà et al. [43]. The values obtained in each reaction were normalized from a standard threshold to minimize variations. The same methodology described by Borja et al. [44] was used to determine the cut-off. The parasite load was expressed by the mean number of parasites per pool triplicate obtained in qPCR reaction. The mean of the parasite load detected in positive pools was calculated as the sum of parasite loads divided by the number of positive pools.

### Calculation of natural infection rate

The *Leishmania* natural infection rate in the sand fly pools was expressed as a minimum infection rate (MIR), calculated as the ratio of the number of positive pools by the total specimens tested x 100, according to Paiva et al. [45].

### Georeferencing and attainment of climatic data

A Global Positioning System (GPS) device, Garmin eTrex HC, was used to georefence the residences included in the study. The software QGIS was used to make the maps of collection sites.

A thermo-hygro-anemometer was employed to attain microclimatic data of temperature and air humidity at the trap locations. These data were measured once per month at the household’s peridomicile during the placement of the traps during the study.

### Influence of climatic variables on monthly fluctuation of *Lu*. *longipalpis* population

The influence of climatic variables on the monthly density fluctuation of *Lu*. *longipalpis* was evaluated along each one of both one-year periods on the beachfront and central areas. Neighborhoods that were highlighted by its higher sand fly density such as Monte Gordo, Barra de Jacuípe, Jauá and Parque Verde II in the first period of the collection were also evaluated isolatedly. Monthly mean values of temperature (°C), air humidity (%) and rainfall (mm3) referring to the first collection year were taken into account for the analysis.

### Statistical analysis

Data banks were created on Epi Info, comprising epidemiological, environmental, bioclimatic and sand fly density data. The correlation between the mean parasite load and the number of sand flies captured per month in each neighborhood was evaluated using Spearman coefficient, it was considered significant when p < 0.05. The correlation between the bioclimatic variables and sand fly population density was evaluated using the Spearman coefficient, it was considered significant when p < 0.05.

## Results

### Sand fly collection

A total of 7,116 sand flies were collected, being 5,745 in period 1 and 1,151 in period 2. Morphological analysis of the sand fly fauna allowed the species identification of 6,321 specimens. In period 1, five species were identified with the predominance of *Lu*. *longipalpis* (98%), followed by *Lutzomyia sallesi* (0.8%), *Lutzomyia evandroi* (0.4%), *Lutzomyia whitmani* (0.2%) and *Lutzomyia choti* (0.1%) and specimens belonging to cortelezzii complex (0.1%), only identified by genus. For the period 2, 1,002 sand flies were identified demonstrating the same predominance of *Lu*. *longipalpis* (99.9%) (Table 1).

**Table 1:** Number of identified sand flies stratified by neighborhoods of Camaçari-BA municipality in the first and second collection periods.

Captured species	First period										Second period				
	Beachfront area			Central area							Beachfront area			Central area	
	MG	JC	JA	PR	VH	NV	PV	PH	JL	Total N (%)	MG	JA	JC	MA	Total N (%)
*Lutzomyia longipalpis*	3,999	482	604	22	2	2	108	1	15	5,235 (98.4)	43	564	394	-	1,001 (99.9)
*Lutzomyia sallesi*	-	-	11	-	2	31	-	-	-	44 (0.8)	-	-	-	-	-
*Lutzomyia evandroi*	16	-	1	-	3	-	1	-	-	21 (0.4)	-	-	-	-	-
*Lutzomyia whitmani*	-	-	1	-	-	-	8	-	1	10 (0.2)	-	-	-	-	-
*Lutzomyia choti*	2	-	-	-	-	2	1	-	-	5 (0.1)	-	-	-	1	1 (0.1)
*Cortellezzi sp*.	4	-	-	-	-	-	-	-	-	4 (0.1)	-	-	-	-	-
**Total**	**4,021**	**482**	**617**	**22**	**7**	**35**	**118**	**1**	**16**	**5,319 (100)**	**43**	**564**	**394**	**1**	**1,002 (100)**

MG: Monte Gordo, JC: Barra de Jacuípe, JA: Jauá, PR: Parque Real Serra Verde, VH: Verde Horizonte, NV: Nova Vitória, PV: Parque Verde II, PH: Phooc III, JL: Jardim Limoeiro, MA: Machadinho.

**Fig 2:**
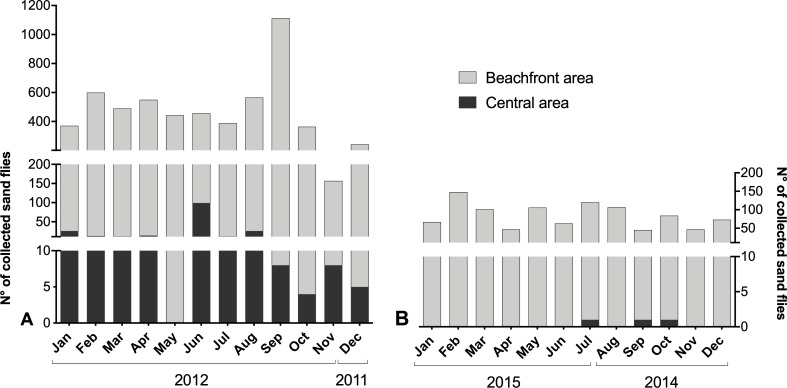
Sand fly monthly distribution in neighborhoods of the central and beachfront areas in the period 1, between December 2011 and November 2012 (A), and in the period 2, between August 2014 and July 2015 (B).

The most significant proportion of the captured sand flies were registered at the beachfront area (Fig 2). In the period 1, 99.6% were captured at this area and 0.4% at the neighborhoods of the central area, while in the period 2, around 100% of the sand flies were captured at the beachfront area. Monte Gordo, Jauá and Barra de Jacuípe had the most significant proportion of collected specimens in period 1, 76%, 12%, and 9% respectively. For the period 2, the collections were also higher in Jauá (56%) and Barra de Jacuípe (39%).

Regarding the monthly fluctuation of *Lu*. *longipalpis* population in the studied area, the highest collection was observed in September 2012 (1109 sand flies) and the lowest in November 2012 (154 sand flies) in the period 1 (Fig 2A), whereas in the period 2 the highest density was observed in February 2015 (371 sand flies) and the lowest in September 2014 (111 sad flies) (Fig 2B). In respect to the sand flies endophilic and exophilic behavior, the sand flies were predominantly collected in the peridomicile in both collection periods (S1 Table).

### Natural infection of *Lu*. *longipalpis*

All pools tested positive for *Lu*. *longipalpis* periodicity control gene by qPCR. The presence of *L*. *infantum* DNA was detected in 60 pools among 74 evaluated, in the period 1, indicating natural infection in the sand fly population with a MIR of 9.6% at the beachfront area (Table 2). The neighborhoods that presented positivity for *L*. *infantum* in captured sand flies were Monte Gordo, Barra de Jacuípe and Jauá, located at the beachfront area (Fig 1). Monte Gordo presented the majority of sand flies captured and positive pools, but MIR in Barra do Jacuípe (14.3%) was higher than in Monte Gordo (9.3%).

**Table 2:** *Lu*. *longipalpis* positive pools and minimal infection rates distributed by month and neighborhoods of the beachfront area of Camaçari-BA municipality in both collection periods.

Study period		Monte Gordo			Barra de Jacuípe			Jauá		
		Sand flies	Pools +/Total	MIR	Sand flies	Pools +/Total	MIR	Sand flies	Pools +/Total	MIR
2011	Dec	23	3/3	13.0	-	-	-	-	-	-
2012	Jan	20	2/4	10.0	-	-	-	-	-	-
	Feb	38	5/5	11.4	7	1/1	14.3	-	-	-
	Mar	30	2/3	6.7	3	1/1	33.3	4	1/1	25.0
	Apr	50	5/5	10.0	9	1/1	11.1	-	-	-
	May	43	3/5	7.0	-	-	-	4	0/1	-
	Jun	37	1/4	2.7	-	-	-	-	-	-
	Jul	3	1/1	33.3	-	-	-	-	-	-
	Aug	-	-	-	-	-	-	-	-	-
	Sep	218	20/23	9.2	-	-	-	10	0/1	-
	Oct	90	9/9	10.0	-	-		4	1/1	16.7
	Nov	7	1/1	14.3	13	3/3	23.1	-	-	-
**Total period 1**		**559**	**52/63**	**9.3**	**42**	**6/7**	**14.3**	**22**	**2/4**	**9.0**
2014	Aug	-	-	-	10	1/2	10.0	14	0/1	7.1
	Sep	-	-	-	2	1/2	50.0	2	1/2	50
	Oct	-	-	-	3	1/1	33.3	6	1/4	16.7
	Nov	-	-	-	28	3/7	10.7	2	0/1	-
	Dec	-	-	-	1	0/1	100.0	36	2/7	5.6
2015	Jan	1	0/1	-	9	0/2	-	3	0/1	-
	Feb	-	-	-	2	0/1	-	5	0/1	-
	Mar	-	-	-	-	-	-	10	0/1	-
	Apr	-	-	-	-	-	-	-	-	-
	May	-	-	-	1	1/1	100.0	3	1/1	33.3
	Jun	9	2/2	22.2	4	1/1	25.0	2	1/1	50.0
	Jul	-	-	-	-	-	-	13	1/1	7.7
**Total period 2**		**10 **	**2/3**	**20.0**	**60**	**8/18**	**13.3**	**96**	**7/21**	**7.3**

For the period 2, 17 out of 42 pools tested positive for the presence of *L*. *infantum* DNA, distributed in Jauá, Barra do Jacuípe and Monte Gordo (Table 2), all located at beachfront area showing a MIR of 10.2%. Barra do Jacuípe presented the majority of positive pools, wherein the MIR was 13.3% lower than Monte Gordo (20.0)%. Natural infection was not detected in sand flies from the central area in both periods.

The natural infection of *Lu*. *longipalpis* was evaluated in all months of the study, except for August 2012 in period 1. *Leishmania* infection was observed in sand fly pools in all evaluated months in period 1. In period 2, the positivity was detected in sand fly pools in May, June, July, August, September, October, November and December (Table 2).

**Fig 3:**
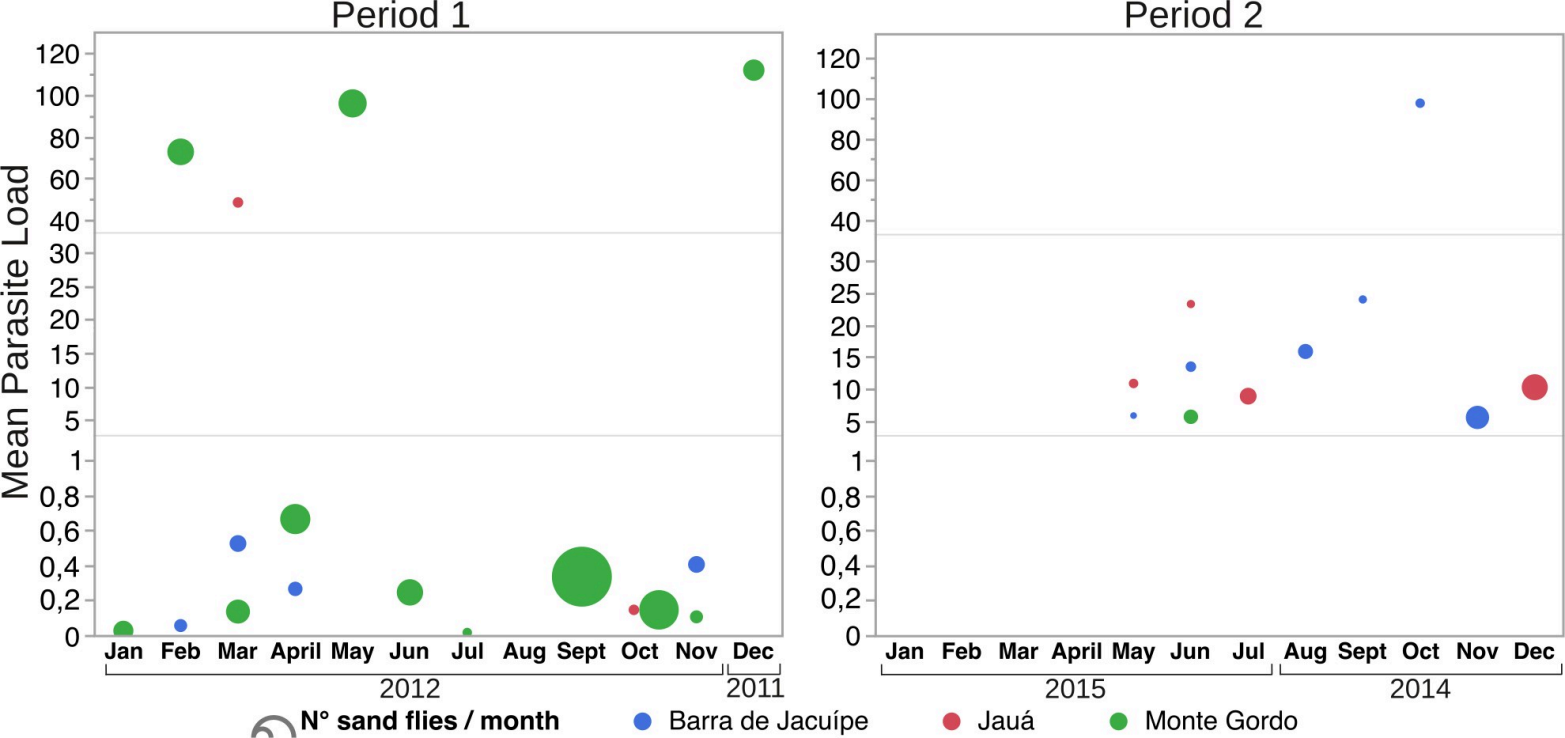
Mean parasite load detected in positive pools per month from each neighborhood. (A) in period 1; (B) in period 2. Dot size represents the number of sand flies captured.

The mean parasite load of the positive pools was constantly low during both studied periods as seen in Fig 3, only three out of 60 positive pools had higher parasite load compared to the remaining ones in Monte Gordo in the first period (February, May and December) and one pool out of 17 in Barra de Jacuipe in the second period (October). The number of sand flies captured per month did not correlate with the mean parasite load.

### Influence of bioclimatic factors on *Lu*. *longipalpis* population

**Fig 4:**
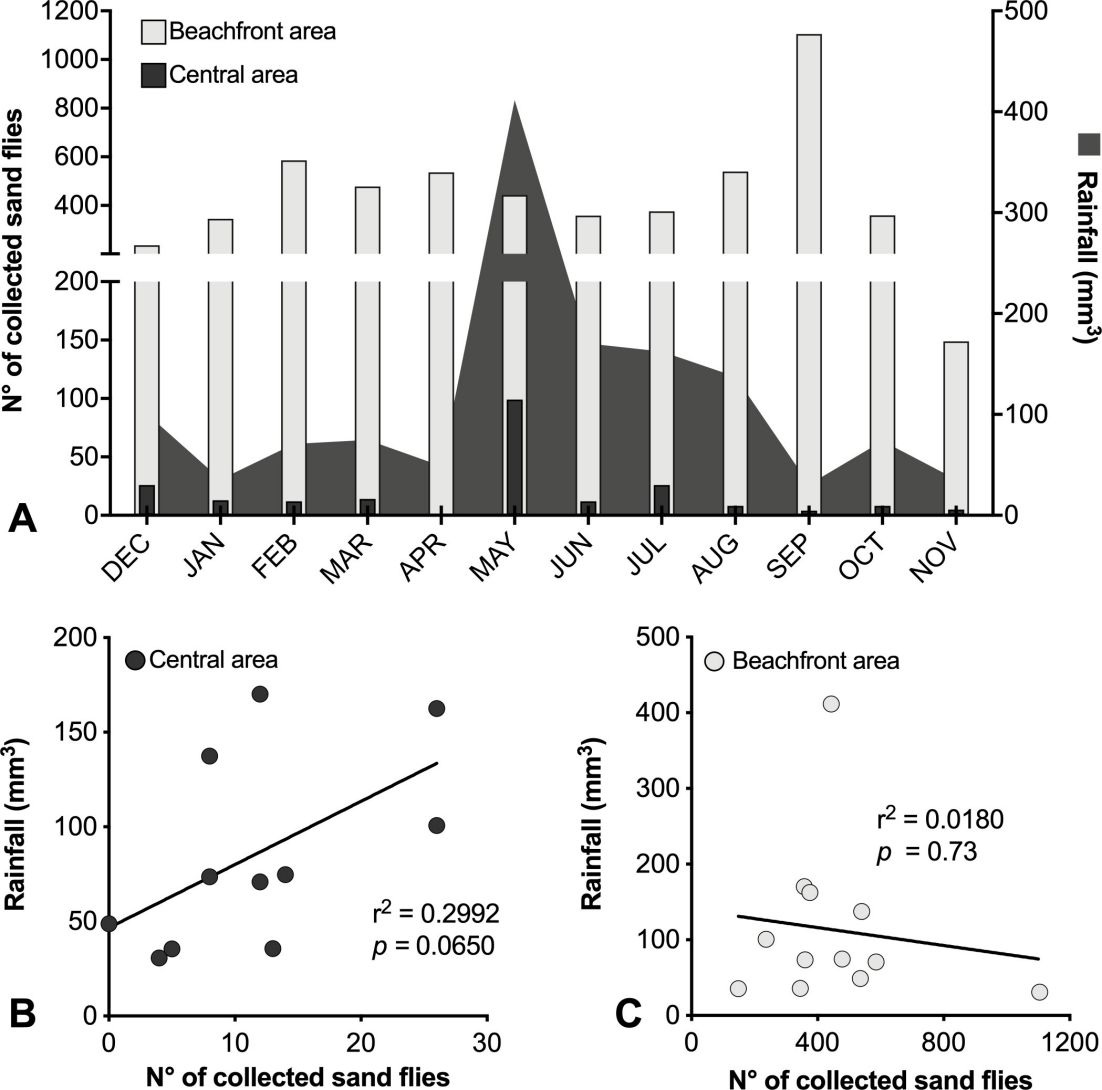
Evaluation of the influence of monthly rainfall on sand flies monthly captured in the Central Area. (A) Distribution of the mean monthly rainfall (mm3) concerning sand flies captured; (B) Correlation between the sand flies captured in central area and the mean monthly rainfall (mm3) (C) Correlation between the sand flies captured in beachfront area and the mean monthly rainfall (mm3) (*r*^*2*^, p: Spearman coefficient).

No correlation was found between any climatic factors and *Lu*. *longipalpis* monthly density in both areas, neither in the aggregated analysis nor looking at specific sites (Fig 4).

### Household characterization

There were 33% of households presenting previous occurrence of CVL cases in period 1 and 44% in period 2. In addition, 47% and 13% presented CVL cases in the neighboring houses in periods 1 and 2 respectively. In 60% of houses in period 1 someone in the past presented VL and 6.7% presented VL cases in the neighboring houses. In period 2, 6.3% of households presented previous VL cases in the same house and 18.8% in the neighboring houses.

Households evaluated in both periods in central and beachfront area did not present statistical differences in their epidemiological characteristics. All of them presented vegetation in their backyard and surrounding area as well as organic matter, such as garbage, decomposing leaves and fruits, construction debris, animal feces and others. Thirty percent of houses in both periods did not have sewage system.

## Discussion

The present study reported, in two moments, the predominance of the species *Lu*. *longipalpis* in the sand fly fauna of Camaçari, in which its natural infection with *L*. *infantum* was determined in neighborhoods of the beachfront area, and aimed to elucidate the influence of bioclimatic factors on the sand fly population.

Our findings demonstrate that although a diverse sand fly fauna, in period 1, has been registered in the investigated areas, the species *Lu*. *longipalpis* predominated in both investigated periods during all months of the study. These data are consistent with findings of similar entomological studies performed in other Brazilian endemic cities for visceral leishmaniasis [46, 47, 48]. *Lu*. *longipalpis* distribution in Camaçari demonstrated the predominance of this vector in neighborhoods located at the beachfront area, especially in the peridomicile of households in Monte Gordo and Jauá. The collection of *Lu*. *longipalpis* in areas close to the sea is an uncommon finding that has been poorly reported. Thus, it strengthens the idea that this vector has high adaptability to different biomes, being also present in environments such as coastal plains with pre-coastal vegetation which provides little subsidy for the formation of ecotopes for reproduction due its microclimatic characteristics [3, 16, 49, 50].

During period 1, two VL cases were identified in Monte Gordo (beachfront area), one case in 2011 and another one in 2012. There were vector control actions in Monte Gordo after VL cases, resulting in a reduction of vector population. The difference observed in Table 1, when the density of captured *Lu*. *longipalpis* in Monte Gordo was lower in the second period in comparison with the first one, should have occurred due the vector control. Additionally, there was a chikungunya outbreak in Camaçari during period 2, which lead to the usage of insecticide spraying to reduce mosquitos that might have influenced the lower collection of sand flies in this period when compared to period 1.

In the present study, it is worth highlighting the capture of *Lu*. *sallesi* due to its importance related to the capability to be naturally infected with *L*. *infantum*, as shown in other studies, suggesting the need for monitoring this species in Camaçari [35]. Other authors emphasize the importance of constant monitoring of naturally infected sand fly species, whether they are known vectors or novel species involved in the transmission of leishmaniasis in endemic areas [28, 33, 35, 51, 52, 53, 54].

Our results demonstrated for the first time the prevalence of *L*. *infantum* infection in the *Lu*. *longipalpis* population in Camaçari. The MIR found in the first and second period (9.6 and 10.2%, respectively) corroborates to the findings of other studies, which demonstrated rates ranging from 0.2% to 10.7% in sand fly populations from other visceral leishmaniasis endemic areas [55, 56, 57, 58, 59, 60, 61]. MIR was similar in both periods demonstrating the sustainability of *Lu*. *longipalpis* populations potentially capable of infecting susceptible reservoirs, over the years in Camaçari municipality. MIR were higher than other studies [56, 62, 63] may be due to the higher sensitivity of the qPCR in comparison with the convenctional PCR used in previous entomological studies [30, 60, 64]. Such higher MIR was also detected using *Leishmania* kDNA-qPCR in a recent study, which detected a MIR of 65% in pools of engorged *Lu*. *longipalpis* females colleted in Rio Verde de Mato Grosso municipality [65]. Thus, a qPCR-based approach, used in the present work, may lead to a MIR closer to the *L*. *infantum* true prevalence in the *Lu*. *longipalpis* population, due to its higher sensitivity.

The identification of female *Lu*. *longipalpis* in all seasons during both collection periods, points out to the possible occurrence of transmission during the whole year, corroborating studies conducted in other endemic areas under distinct conditions [66, 67]. Moreover, throughout the study, in both periods, the parasite load was low and did not vary in the evaluated months despite the number of collected sand flies. Thus, this finding suggests that the risk of infection is the same during the whole year regardless of sand fly density.

Detection of *L*. *infantum* infection in samples from the *Lu*. *longipalpis* population, high prevalences of CVL and VL cases in Camaçari was observed in the same areas evaluated in two different moments. In both periods of the study, the highest CVL prevalences, the occurrence of VL cases, the number of collected sand flies and infection rate of vectors was observed in locations at the beachfront area. These findings corroborated with other prevalence studies [15, 16] and showed that the coastal area of Camaçari presents a high risk of CVL and VL.

The most significant proportion of captured *Lu*. *longipalpis* with evidence of natural infection was detected in the beachfront area, especially in Monte Gordo, Barra de Jacuípe and Jauá in contrast with the central area. This finding was not related to climatic factors or household characteristics such as presence of vegetation, hygiene conditions in the peridomicile and breeding of livestock and/or domestic animals. In this study the factors related to this difference was not identified. A more significant proportion of *Lu*. *longipalpis* was detected in the peridomicile, which is in accordance with results observed by other authors [17, 18, 20, 66, 68, 69]. Households studied in the first period were not the same as in the second period, as well as localities representing central area, but this difference possibly did not interfere in the analysis comparing central and beachfront areas. This is due to absence of statistical differences in household characterization between them comparing both periods.

The influence of climatic factors on *Lu*. *longipalpis* population was evaluated in the present study during the first collection period, and our results indicated that in the study area sand fly population density was not influenced by temperature, humidity and rainfall variations. Several authors found a correlation with climatic factors, observing a higher prevalence of *Lu*. *longipalpis* during the rainy season [70, 71, 72, 73, 74], and other ones demonstrated a preference of this species to dry and rocky areas [75, 76, 77]. The lack of correlation among climatic factors and sand fly density in the beachfront area, where more than 96% of sand flies were captured, and the limited correlation found in central area indicated that other factors could be more important in modulating the sand fly population than climatic factors in the area studied. This finding probably occurs because in this area temperature and humidity does not vary along the year.

Regarding the temperature, there was no significant effect of its variation on *Lu*. *longipalpis* population, not being this fact a determinant factor of this species in monthly fluctuation in this municipality, as demonstrated by other authors concerning other regions of Brazil [66, 70, 78]. It is worth to highlight that the annual temperature cycle in Camaçari has a small range of variations, in average between 23°C and 27°C, which can justify the absence of correlation of this climatic factor. In other studies, similar results were found in other northeastern municipalities where the annual temperature cycle has likewise a small range of variation [79, 80]. In localities with such small temperature variation, changes in the rainfall and humidity should have a larger influence on the fluctuation of *Lu*. *longipalpis* population, as demonstrated by other authors’ findings [72, 73, 74, 80]. However, our findings did not found humidity influence, possibly because humidity also has a small range of variation in Camaçari as well as coastal area, humidity remains high during the whole year.

Additionally, our more significant proportion of *L*. *infantum* positive sand flies found in the beachfront area denies what was proposed by Nieto and collaborators [76] niche modeling. It was shown in their work that the coastal area of Bahia has a lower risk for visceral leishmaniasis when compared to the central Caatinga region. Curiously, there was an outbreak of the disease in Camaçari after this study with both canine and human cases [16, 22]. The expansion to coastal areas has occurred due to recent environmental changes that were done in the beachfront of Camaçari for the construction of touristic and vacation complexes. The rapid urbanization of this location brought new human inhabitants who are used to breed dogs, chickens, and livestock in the peridomicile, providing breeding sites and blood source for *Lu*. *longipalpis* population.

In sum, there was no correlation between bioclimatic factors and sand fly density, being *Lu*. *longipalpis* the most prevalent sand fly species in Camaçari during both studied periods. Furthermore, the parasite load in *Lu*. *longipalpis* was predominantly low and did not fluctuate during the whole year despite the number of collected sand flies. These findings emphasize the high risk of *Leishmania* transmission in Camaçari regardless of the season and point out to the urgency for vector control actions, diminishing the number of CVL and VL cases especially in the neighborhoods in the municipalities beachfront area.

## Supporting information

S1 Table The density of sand flies captured in each household’s investigated sector according to the identified species in Camaçari-BA municipality between December 2011 and November 2012 and between August 2014 and July 2015. (DOCX)
