# Supplementary material for: Natural infection by Leishmania infantum in the Lutzomyia longipalpis population of an endemic coastal area to visceral leishmaniasis in Brazil is not associated with bioclimatic factors
Source: PLoS Negl Trop Dis. 2019 Aug 26;13(8):e0007626. doi: 10.1371/journal.pntd.0007626 (PMC6730935; doi:10.1371/journal.pntd.0007626)
Supplement: S1 Table — (DOCX) [file pntd.0007626.s001.docx]

**S1 Table**

|  | **Captured species** | **Intradomicile** | | **Peridomicile** | | **Total** | **%** |
| --- | --- | --- | --- | --- | --- | --- | --- |
| **Study period** |  | **male** | **female** | **male** | **female** |  |  |
| 1 | *Lutzomyia longipalpis* | 256 | 58 | 3,990 | 931 | 5,235 | 98.4 |
|  | *Lutzomyia sallesi* | 2 | 1 | 1 | 40 | 44 | 0.8 |
|  | *Lutzomyia evandroi* | 4 | 7 | 5 | 5 | 21 | 0.4 |
|  | *Lutzomyia whitmani* | 2 | - | 1 | 7 | 10 | 0.2 |
|  | *Lutzomyia choti* | 1 | - | 3 | 1 | 5 | 0.1 |
|  | *Cortellezzi sp.* | - | - | 4 | - | 4 | 0.1 |
|  | **Subtotal (%)** | **265 (4.9)** | **66 (1.2)** | **4,004 (75.2)** | **984 (18.5)** | **5,319** | **(100)** |
| 2 | *Lutzomyia longipalpis* | 50 | 7 | 872 | 221 | 1,150 | 99.9 |
|  | *Lutzomyia choti* | 1 | - | - | - | 1 | 0.1 |
|  | **Subtotal (%)** | **51**  **(4.4)** | **7**  **(0.6)** | **872**  **(75.8)** | **221**  **(19.2)** | **1,151** | **(100)** |
|  | **Total (%)** | **389 (6.1)** | | **6081 (93.9)** | | **6470(100)** |  |
